# Surgeons' Experiences After Surgical Errors: Insights From a Urology Department

**DOI:** 10.1002/hsr2.71942

**Published:** 2026-02-26

**Authors:** Ehsan Sepehran, Mohsen Amjadi, Neda Kabiri, Seyed Faraz Mortazavi, Shabnam Ghasemyani, Arash Mohagheghi, Farzin Soleimanzadeh

**Affiliations:** ^1^ Department of Urology, Faculty of Medicine Tabriz University of Medical Sciences Tabriz Iran; ^2^ Research Center for Evidence‐Based Medicine: A JBI Centre of Excellence, Faculty of Medicine Tabriz University of Medical Sciences Tabriz Iran; ^3^ Medical Philosophy and History Research Center Tabriz University of Medical Sciences Tabriz Iran; ^4^ Student Research Committee Tabriz University of Medical Sciences Tabriz Iran; ^5^ Department of Health Service Management, School of Health Management and Information Sciences Iran University of Medical Sciences Tehran Iran; ^6^ Research Center of Psychiatry and Behavioral Sciences Tabriz University of Medical Sciences Tabriz Iran

**Keywords:** experiences, qualitative study, surgical errors, urology

## Abstract

**Background and Aims:**

Surgical practice is rewarding but also challenging, and adverse patient events can deeply affect surgeons. In this study, we explored how such events influence surgeons' personal well‐being, emotional state, and professional performance.

**Methods:**

This qualitative descriptive study was conducted using semi‐structured interviews with 13 urology faculty members and residents at Tabriz University of Medical Sciences, Tabriz, Iran. Data were analyzed using thematic analysis.

**Results:**

Thirteen participants, including four faculty members and nine residents, were interviewed to understand their experiences and coping strategies with surgical errors. Types of surgical errors in the field of urology included technical, cognitive, clinical decision‐making, unintentional, fatigue‐related, diagnostic, and equipment‐ or environment‐related errors. Six themes were emerged regarding participants' experiences including psychological and emotional impact, impact on professional practice, social support and relationships, external contributing factors, access to psychological support, and legal consequences.

**Conclusion:**

Our study showed that adverse events in surgery affect surgeons both personally and professionally. Supporting surgeons through these experiences, by institutional programs such as establishing formal counseling centers, can reduce stress and enhance learning. Recognizing that mistakes are part of surgical practice and using them as opportunities for growth can benefit both surgeons and their patients.

## Summary

- All healthcare providers can do mistakes, knowing how to manage personal and psychological impacts of these errors is important.
- Surgical errors included technical, cognitive, clinical decision‐making, unintentional, fatigue‐related, diagnostic, and equipment‐ or environment‐related.
- Institutional programs such as establishing formal counseling centers within or outside of the hospital, can help surgeons manage their stress.

## 1 Introduction

Unintentional medical errors are estimated to be the third leading cause of death in the United States, and human error among healthcare professionals remains a persistent concern [1]. Although medicine is a healing profession and patient care and safety are essential priorities, human imperfection makes errors inevitable in complex healthcare environments and clinical practices [2]. These errors can have negative consequences not only for patients but also for physicians, nurses, and healthcare institutions [3, 4]. Contributing factors include emergency situations, severe patient conditions, the complexity of diagnostic and therapeutic methods, and the increasing use of advanced procedures and equipment [5].

Despite their frequency, medical errors are generally underreported, partly due to their subtlety and difficulty of recognition [6]. One major challenge faced by physicians is how to disclose such errors to patients. Disclosure raises concerns regarding the physician‐patient relationship, potential career challenges, and the risk of litigation [7, 8].

Although patients are the primary victims of medical errors, evidence suggests that healthcare providers are also deeply affected [9]. The concept of “secondary victim” has been introduced to describe caregivers who are psychologically harmed by errors in which they were involved and for which they feel personally responsible. Secondary victims often experience self‐doubt, loss of confidence in their clinical skills, and feelings of failure, which can impact healthcare professionals [10].

Healthcare providers who perceive themselves as responsible for errors may suffer from guilt, hopelessness, fear, shame, anxiety, depression, job stress, post‐traumatic stress disorder, and even suicidal ideation [9]. Errors may also result in long‐term consequences such as impaired concentration, burnout, poor memory, decreased self‐confidence, and diminished work performance and safety [11], sometimes leading physicians to leave the profession entirely. Physicians also report that errors negatively affect their job satisfaction, sleep quality, relationships with colleagues, and overall sense of self‐worth. For example, a study of 184 physician assistants found that committing an error was associated with reduced quality of life and increased rates of depression and burnout [12].

Although counseling and support are routinely provided to patients and their families, the psychological burden on physicians themselves is often overlooked. A review of the literature indicates that this issue remains underexplored worldwide [11], with limited recent attention. Therefore, the present study was designed to investigate the psychological experiences and challenges faced by faculty members and residents in the Urology Department of Tabriz University of Medical Sciences following surgical errors.

## 2 Methods

### 2.1 Study Design and Setting

This qualitative descriptive study was conducted in the Urology Department of Tabriz University of Medical Sciences, Tabriz, Iran to explore the psychological experiences and professional challenges of surgeons following surgical errors. Participants including academic staff and urology residents were selected using purposive sampling to ensure diverse perspectives regarding age, gender, and professional experience.

### 2.2 Data Collection

Data were collected through semi‐structured interviews. The interview guide included questions which covered the description of psychological and professional condition after error, preference of surgeon whether to continue the rest of the surgery or not, and whether to do the same surgery in the next days, need for speak others or need for any support system. Participants were asked to reflect on surgical errors throughout their entire professional careers. Participants were asked to describe recent incidents they could recall in detail, in order to facilitate discussion and provide concrete illustrations. All interviews were conducted face‐to‐face, in a private room in the urology department by a trained male medical student under the supervision of an experienced qualitative researcher. Interviews were piloted in the first three participants. The interviews ranged from 20 to 33 min. The objectives of the research and the reasons and interests of the research topic were first described to the participants. We continued data collection until we reached conceptual depth.

### 2.3 Data Analysis

All interviews were audio‐recorded after informed consent was achieved and transcribed verbatim. Also, field‐notes were taken during the interviews. Data were analyzed using thematic analysis of Braune and Clarke [13], which included six phases of familiarization with data, generation of initial codes, search for themes, review of themes, defining and naming themes, and producing the report. Data analysis was conducted by two authors and checked and finalized by all authors. We managed the data using the qualitative data analysis software MAXQDA v10 (2011). This study was conducted and reported following the Consolidated Criteria for Reporting Qualitative Research (COREQ) to ensure transparency and rigor in the reporting of qualitative methods and findings.

## 3 Results

### 3.1 Participant Characteristics

A total of 13 participants (4 faculty members and 9 residents) took part in the study. In this study, faculty members refer specifically to consultant‐level urologists with clinical and surgical responsibilities, ensuring that insights reflect direct surgical experience rather than non‐clinical perspectives. The participants included 10 males and 3 females. Table 1 shows the main characteristics of participants.

**Table 1:** Main characteristics of participants.

Participant ID	Gender	Age	Role	Years of experience
P1	Male	40	Faculty members	4
P2	Female	47	Faculty members	7
P3	Male	62	Faculty members	35
P4	Male	47	Faculty members	10
P5	Male	30	Resident	3
P6	Female	32	Resident	3
P7	Male	29	Resident	2
P8	Male	30	Resident	3
P9	Male	30	Resident	4
P10	Male	30	Resident	4
P11	Male	38	Resident	3
P12	Female	34	Resident	3
P13	Male	29	Resident	2

### 3.2 Types of Surgical Errors

Based on the interviews, participants identified a range of errors they had experienced during surgical practice. The reported errors were categorized into seven major types of technical error, cognitive error, clinical decision‐making error, unintended error, error due to fatigue or psychological stress, diagnostic error, and equipment error (Table 2).

**Table 2:** Types of surgical errors reported by participants.

Type of surgical error	Description/Example
Technical error	Mistakes in surgical technique, such as injury to anatomical structures or errors in incision or suturing.
Cognitive error	Inability to anticipate or make appropriate decisions in a specific clinical situation.
Clinical decision‐making error	Incorrect patient selection for surgery or overlooking risk factors.
Unintended error/complication	Adverse events despite correct technique, for example, postoperative infection or urinary leakage.
Error due to fatigue or psychological stress	Reduced concentration during surgery because of fatigue, sleep deprivation, stress, or external factors.
Diagnostic/history‐taking error	Incomplete patient history or insufficient understanding of clinical condition.
Equipment/environmental error	Device malfunction during surgery or suboptimal operating room conditions.

### 3.3 Experiences and Challenges of Surgeons After Error

Thematic analysis of the interviews revealed six major themes regarding the psychological and professional consequences of surgical errors including emotional and psychological reactions, the impact of errors on their clinical performance, the importance of social support, and the influence of external stressors such as fatigue and personal problems, the importance of access to psychological support systems, and concerns about legal repercussions following surgical errors. The identified themes, subthemes, and illustrative quotes are summarized in Table 3.

**Table 3:** Themes, subthemes, and illustrative quotes from participants.

Theme	Subtheme	Illustrative Quote
Psychological and emotional impact	Stress and anxiety	*“I was under so much stress that I almost lost control; it was a terrible situation.”* (P8)
	Mood and sleep disturbance	*“I couldn't sleep for days; my mind was fully occupied, and I had no motivation.”* (P1)
	Guilt and self‐blame	*“The hardest part was the guilt I felt for the complication that happened to the patient.”* (P10)
	Loss of confidence	*“When the professor criticized me in front of others, I lost my self‐confidence.”* (P8)
	Regret and self‐questioning	*“I asked myself why I am even doing surgeries when I don't need the money.”* (P3)
	Hopelessness and discouragement	*“Sometimes after an error, you feel like maybe you are not meant for this profession.”* (P5)
	Hyper‐vigilance/over‐cautiousness	*“Since that event, I double‐check everything many times before and during surgery.”* (P2)
Impact on professional practice	Effect on subsequent operations	*“After that error, I paid much more attention in the next operations.”* (P8)
	Increased awareness and learning	*“The incident raised my awareness instead of making me fearful.”* (P11)
	Attitude toward errors and management	*“We must accept that complications may occur and learn how to manage them.”* (P9)
Social support and relationships	Support from professors and colleagues	*“My professor told me to go home and later reassured me that I wasn't to blame.”* (P12)
	Constructive criticism versus superficial empathy	*“Even criticism was better than hearing ‘don't worry, it's nothing.’”* (P11)
	Family support	*“Talking to my spouse made me feel much better.”* (P4)
	Support from surgical team	*“My professor always says: this won't be your first or last complication; you must live with it.”* (P10)
	Impact on personal relationships	*“When I was scolded, I went home angry and transferred that stress to my family.”* (P13)
External contributing factors	Fatigue and sleep deprivation	*“Sleepiness and fatigue have led me to make mistakes.”* (P13)
	Conflicts before surgery	*“Arguments with OR staff before surgery ruined my focus and caused complications.”* (P7)
	Financial problems	*“I was constantly thinking about financial issues, which distracted me during surgery.”* (P5)
	Personal stressors	*“I was preoccupied, had hand tremors, and my surgeries lacked quality.”* (P6)
	Heavy workload	*“Too many operations in a day, especially at the end, increased the risk of complications.”* (P2)
	Underestimating procedures	*“Considering a procedure as ‘simple’ made me careless and led to errors.”* (P4)
	Incomplete patient history	*“A resident missed the patient's radiotherapy history, and we made the wrong surgical decision.”* (P10)
	Equipment malfunction	*“The endoscope lens was damaged, visibility was poor, but I continued and caused ureteral injury.”* (P5)
Access to psychological support	Need for psychologist/listener	*“It helps a lot if someone just listens and understands the stress of being a surgeon.”* (P7)
	Counseling unit in hospital	*“If we had access to a psychologist at the hospital, especially right after the event, it would help.”* (P7)
	Routine psychological visits	*“Residents should have regular visits to psychologists once or twice a year.”* (P13)
	Supportive work culture	*“If we had someone in our group to talk to, it would be very helpful.”* (P11)
Legal consequences	Fear of lawsuits	*“Even though the patient was not harmed, they complained, and I was summoned to court.”* (P4)
	Concerns about financial compensation	*“The legal aspects and the possibility of paying blood money are very stressful.”* (P4)

## 4 Discussion

A total of 13 participants (4 faculty members and 9 residents) took part in the study. Types of surgical errors in the field of urology included technical, cognitive, clinical decision‐making, unintentional, fatigue‐related, diagnostic, and equipment‐ or environment‐related errors.

Our results showed that surgical errors had significant psychological and emotional impacts, such as increased stress, mood and sleep disturbances, guilt, reduced self‐confidence, regret, and heightened caution in subsequent procedures. A similar study by O'Meara et al. in 2025 was conducted to assess personal impacts of surgical errors in surgeons, and revealed that 89% of respondents experienced negative psychological symptoms such as anxiety, guilt, and low mood following adverse events. Also, 67% felt that their training did not adequately prepare them for the personal impact of such events. However, many found informal peer and family support beneficial and expressed openness to implementing formal support structures. This study highlights that the significant emotional toll surgical errors can have on healthcare professionals and underscores the need for institutional support systems to assist in coping with these challenges [14]. Also, according to a recent review on the impact of adverse events on surgeons, such events significantly affect physical and mental health, psychological wellbeing, and professional performance. Surgeons often feel unprepared for these consequences and under‐supported when they occur [15]. Srinivasa et al. Srinivasa et al., [16] conducted a systematic review examining how patient complications affect surgeons' well‐being, including emotional outcomes, coping strategies, and support mechanisms and identified common emotional responses such as anxiety, guilt, sadness, and shame, as well as interference with professional and personal life, similar to the stress, guilt, decreased self‐confidence, and emotional burden reported by participants in our study.

Results of our study indicated that social support from supervisors, colleagues, and families mitigated negative effects. These results are consistent with a recent mixed‐methods study by Ginzberg et al. Ginzberg et al., [17], which showed that structured support, especially the opportunity to discuss events with attending physicians or peers, was the most helpful coping mechanism. Additional literature further highlights the range of coping strategies surgeons employ after surgical errors. Koirala et al. Koirala et al., [18] reported that surgeons use techniques such as positive self‐instruction, distraction, and emotional regulation, although not all strategies are equally effective, underscoring the importance of formal support systems. Furthermore, D'Angelo et al. D'Angelo et al., [19] introduced the STOPS framework (Stop, Talk, Obtain help, Plan, Succeed) as an evidence‐based intraoperative coping curriculum that can train surgeons and residents to manage errors effectively.

Our results showed that external factors such as fatigue, high workload, and financial or personal issues were identified as the main contributors to surgical errors. In line with these findings, another study indicated that external factors such as workload pressure, resource limitations, and high expectations from patients and their families significantly impacted surgeons' psychological and emotional well‐being, and lead to their stress, anxiety, and professional burnout [20]. These factors reduce surgeons' concentration and precision, and increase their stress. Limited resources or lack of support at work can worsen this situation. Managing these external factors through better workload planning, institutional support systems, and access to mental health resources is essential for both surgeon and patient.

## 5 Conclusion

Our study disclosed that the happening of adverse events in surgery has consequences on both personal and professional lives of surgeons as a second victim. On the other hand, these errors improved surgeons' concentration, awareness, and attitudes toward error management. Participants reported employing various coping mechanisms, such as seeking support from colleagues and supervisors, reflective practice, and structured strategies to manage stress and maintain performance. One of the essential services that hospitals can provide in order to lessen the upcoming errors is establishing formal counseling centers within or outside of the hospital and make it obligatory for surgeons to receive counseling services constantly. Accepting the reality that errors are part of surgical practice and using them as opportunities for growth can benefit both surgeons and their patients.

### 5.1 Study Limitations

One limitation of this study is the relatively small sample size, particularly the limited number of faculty members, which may affect the generalizability of the findings; however, this is inherent to qualitative research, where the focus is on gaining in‐depth understanding of participants' experiences rather than statistical representativeness. Another limitation is that the study was conducted in a single urology department, which may limit the transferability of findings to other surgical specialties or healthcare settings.

### 5.2 Policy Implications

Hospitals should implement structured counseling programs for surgeons and institutionalize these programs as a routine and required part of surgical practice. Furthermore, institutional policies should explicitly recognize surgeons as “second victims” of adverse events and integrate support mechanisms into quality and safety protocols.

## Author Contributions

Conceptualization: Ehsan Sepehran, Mohsen Amjadi, Neda Kabiri. Data curation: Seyed Faraz Mortazavi, Neda Kabiri. Formal analysis: Neda Kabiri. Funding acquisition: Not applicable. Investigation: Seyed Faraz Mortazavi. Methodology: Neda Kabiri, Farzin Soleimanzadeh. Project administration: Ehsan Sepehran, Mohsen Amjadi, Arash Mohagheghi. Resources: Seyed Faraz Mortazavi. Software: Neda Kabiri. Supervision: Ehsan Sepehran, Mohsen Amjadi. Validation: Arash Mohagheghi. Visualization: Not applicable. Writing – original draft preparation: Shabnam Ghasemyani, Seyed Faraz Mortazavi, Neda Kabiri. Writing – review and editing: all authors.

## Funding

The authors received no specific funding for this work.

## Ethics Statement

The Ethics Committee of the Tabriz University of Medical Sciences approved this study (IR.TBZMED.FMD.REC.1403.016). We obtained signed informed consent from all participants. All participants were free to leave the study at any time. Participants' quotes were anonymized by removing their information.

## Consent

We obtained signed informed consent from all participants.

## Conflicts of Interest

The authors declare no conflicts of interest.

## Transparency Statement

The lead author Neda Kabiri, Seyed Faraz Mortazavi affirms that this manuscript is an honest, accurate, and transparent account of the study being reported; that no important aspects of the study have been omitted; and that any discrepancies from the study as planned (and, if relevant, registered) have been explained.

## Data Availability

All data related to study are available in the article.
